# Dysmenorrhea—A Clinical Note

**Published:** 1902-09

**Authors:** J. E. Dearden

**Affiliations:** New York City, 136 Lexington Avenue.


### Dysmenorrhea—A Clinical Note.

By J. E. DEARDEN, M. D., New York City.

Bessie K., 19 years of age, consulted me in March, 1900, for dysmenorrhea from which she suffered severely. She began menstruating at 15 years of age. For the past year she has been growing worse so that at the time of consultation she was compelled to spend several days in bed at each period. An examination disclosed no organic trouble. She was put on Hayden's viburnum compound, one teaspoonful in hot water four times daily for five days before the expected menstrual flow, and a teaspoonful every two hours when the flow began.

The second period after treatment she passed in comparative comfort, and after seven months further treatment was abandoned as unnecessary. She has had up to the present time no return of the dysmenorrhea.

I have used this remedy in my practice for a number of years and have been very well satisfied with the results obtained.

1368 Lexington Avenue.

